# An aggravated return-to-work case of organic solvent induced chronic toxic encephalopathy

**DOI:** 10.1186/s40557-018-0232-1

**Published:** 2018-04-27

**Authors:** Sangyun Seo, Jungwon Kim

**Affiliations:** 1 Department of Occupational and Environmental Medicine, Kosin University Gospel Hospital, 34 Amnam-dong, Seo-gu, Busan, 602-702 Republic of Korea; 2 Department of Occupational and Environmental Medicine, Kosin University College of Medicine, Busan, Republic of Korea

**Keywords:** Toxic encephalopathy, Solvents, Occupational diseases, Neurotoxicity syndromes, Return to work

## Abstract

**Background:**

Organic solvent-induced chronic toxic encephalopathy (CTE) is known as a non-progressive disorder that does not progress after diagnosis. The authors present a case those symptoms worsened after continued exposure to organic solvent after returning to work. Because such a case has not been reported in South Korea to the best of our knowledge, we intend to report this case along with literature review.

**Case presentation:**

A 59-year-old man, who performed painting job at a large shipyard for 20 years, was receiving hospital treatment mainly for depression. During the inpatient treatment, severe cognitive impairment was identified, and he visited the occupational and environmental medicine outpatient clinic for assessing work relatedness. In 1984, at the age of 27, he began performing touch-up and spray painting as a shipyard painter. Before that he had not been exposure to any neurotoxic substances. In 2001, at the age of 44, after 15 years of exposure to mixed solvents including toluene, xylene and others, he was diagnosed with CTE International Solvent Workshop (ISW) type 2A. After 7 years of sick leave, he returned to work in 2006. And he repeated return-to-work and sick leave in the same job due to worsening of depressive symptoms. He had worked four times (2006–2010, 2011–2011, 2011–2011, 2016–2017) for a total of 5 years as a shipyard painter after first compensation. During the return-to-work period, the mean values of the mixed solvent index ranged from 0.57 to 2.15, and except for a one semiannual period, all mean values were above the standard value of 1. We excluded other diseases that can cause cognitive impairment like central nervous system diseases, brain injury, psychological diseases and metabolic diseases with physical examinations, laboratory tests, and brain image analysis. And finally, throughout neuropsychological tests, an overall deterioration in cognitive function was identified compared to 2002, and the deterioration types was similar to that often shown in the case of CTE; thus a diagnosis of CTE (ISW) type 3 was made.

**Conclusion:**

This case is showing that CTE can go on with continued exposure to mixed solvents. Appropriate “fitness to work” should be taken to prevent disease deterioration especially for the sick leave workers.

## Background

Chronic toxic encephalopathy (CTE) is a disease resulting from a long-term exposure to low-concentrated organic solvents. The chronic adverse effects of organic solvents on the brain began to be reported in the 1960s. Since the long-term low-concentrated exposure to organic solvents was recognized mainly in Nordic countries in the 1970s, CTE has been considered as an occupational disease [1, 2]. CTE is a disease that can progress from non-specific symptoms to mood disorders such as depression and further to decreased cognitive function and movement disorders [3–5]. The age of diagnosis varies according to the exposure period, and a high incidence is reported among males and specific occupations (e.g., painters, wooden surface finishers, floor layers) [4]. According to International solvent workshop (ISW), CTE can be classified in four stages according to clinical features. Type 1 displays only non-specific symptoms; type 2A is characterized by sustained personality change that involves fatigue, mood and motivation; type 2B displays decreased cognitive function such as memory and concentration; and type 3 is the dementia stage in which global deterioration in intellect and memory is often accompanied by abnormal neurologic signs and neuroradiologic findings [6]. As of 2010, there were a total of 10 workers who had ever received worker’s compensation due to mixed organic solvent-induced CTE: four were identified as type 3; two as type 2B; three as type 2A; and 1 as type 1 [5]. In general, CTE is known as a non-progressive disorder whose symptoms cease progressing or partially recover after diagnosis [7]. However, authors have confirmed that decreased cognitive function in the patient who was already diagnosed with CTE type 2A and received worker’s compensation in 2001. Because in South Korea, no cases have been reported regarding aggravated CTE, we report this case with review of literatures.

## Case presentation

### Patient information

Fifty-nine years old, Male.

### Chief complaint

Severe impairments in neurocognitive functions were identified during the treatment of his depressive disorder.

### Present illness

He was diagnosed with substance-induced depressive disorder in the mental health clinic of our hospital and was hospitalized for treatment. During this hospitalization, broad cognitive impairments were identified in the neuropsychological tests performed in January 2017. He visited the occupational environmental medicine clinic of Kosin University Gospel Hospital as an outpatient for the evaluation of the association with work.

### Past medical history of patient and family

The patient did not have a notable medical history except being hospitalized for herniated lumbar disc in 1997 and noise induced deafness in 1988. He did not have a family history of nervous disease or mental disease. He received worker’s compensation due to substance-induced depressive disorder in 2001.

### Social history

He was married. With regard to the smoking history, the patient began smoking about a pack of cigarettes per week at age 20 and quit smoking after age 40. As for the alcohol history, he said that he drank about a bottle of soju per week from age 20 to 40.

### Occupational history

The patient worked in the restaurant business for four years from age 23, and began working as a shipyard painter at age 27 in 1984. He was employed at the large shipyard D in July 1985. Since then he performed outdoor touch-up painting and spray painting ratio of 50 to 50. According to his statement, in his early working period, he had used inappropriate protection gear such as cotton cloth instead of gas mask. He also mentioned that he often worked overtime (until 10 p.m.), although the regular working hours were from 8 a.m. to 5 p.m. He was diagnosed with depressive disorder resulting from organic solvent intoxication, and it was approved as an industrial accident in 2001. He returned to work in 2006 and worked as a shipyard painter for about four years. Later, he repeatedly alternated between a leave of absence and return-to-work due to worsening of depressive symptoms. His working periods are shown in detail in the Table 1. His total working period as a shipyard painter is about 20 years.

**Table 1:** Work history based on the worker’s statements and work records

Period	Work history
Jun. 1984–Jun. 1985	Performed painting job for another company.
Jul. 1985–Nov. 1997	Employed at D Shipbuilding to perform painting job
Nov. 1997–Jun. 1998	Took a leave of absence due to herniated lumbar disc
Jul. 1998–Nov. 1999	Performed painting job
Nov. 1999–Oct. 2006	Took a leave of absence due to organic solvent-induced depressive disorder
Nov. 2006–Jun. 2010	Performed painting job
Jun. 2010–Sep. 2010	Took a leave of absence
Oct. 2010–Feb. 2011	Performed painting job
Mar. 2011–Jun. 2011	Performed distribution job
Jun. 2011–Nov. 2011	Performed painting job
Nov. 2011–Aug. 2016	Took a leave of absence due to organic solvent-induced depressive disorder
Aug. 2016–Jan. 2017	Performed painting job
Jan. 2017-	Inpatient treatment for depression

### Exposure assessment

**Fig. 1:**
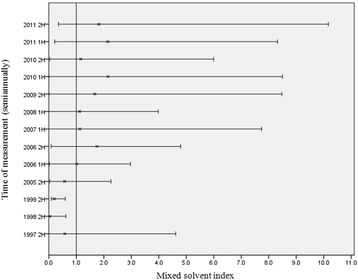
Estimations of mixed solvent exposure. In this graph, horizontal lines are representing minimum and maximum value of mixed solvent index, and ‘X’ are showing mean values of mixed solvent index. The reference level of the mixed solvent index is 1.0

Epidemiological investigation data written by the Occupational Safety and Health Research Institute (OSHRI) were referred to regarding the exposure before 2001, while the results of working environmental monitoring were used with regard to the exposure after his return to work. The mean values of the mixed solvent index (the reference level is 1.0) before 2001 were 0.04 to 0.58, which were within the standard range. However, this result had limitations because the data before 1997 could not be checked. As for the level of exposure to mixed organic solvents at the painting department after his return to work, the mean values of the mixed solvent index were above the standard value during most periods (Fig. 1). There were some changes in the target substance, but common items were isopropyl alcohol (exposure standard 200 ppm), 2-Methyl-1-propanol(50 ppm), n-Butanol(20 ppm), Propylene glycol methyl ether(100 ppm), Methyl isobutyl ketone(50 ppm), Toluene(50 ppm), Ethylbenzene(100 ppm), xylene(100 ppm), trimethylebenzene(25 ppm). A case with more than ten times the standard value was identified on an individual basis, and therefore there is a possibility that the worker of this case was exposed to a considerable amount of mixed solvents.

#### Laboratory tests

Complete blood cell count, urinanalysis, thyroid function tests and liver function tests were performed while the patient was hospitalized, and they showed normal findings. The C-reactive protein value was 0.041, which was within the normal range.

#### Neuroimaging tests and neurophysiological tests

**Fig. 2:**
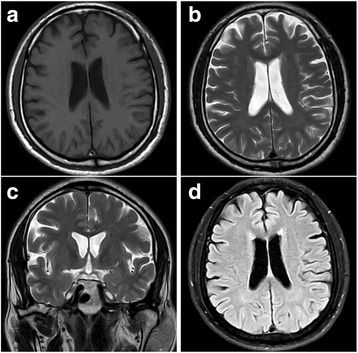
Brain magnetic resonance images (MRI) showed normal anatomy of brain without any mass or atrophic lesions. **a** T1-weighted axial image. **b** T2-weighted axial image. **c** T2-weighted coronal image. **d** T2-FLAIR axial image

Brain magnetic resonance imaging (MRI) was performed in May 2017; the images showed no structural abnormalities, such as neoplasm and traumatic injury, as well as atrophy. Although only It was confirmed by medical record, MRI performed in 2002 and 2006 also showed no notable findings, Again, according to his past medical records, the electroencephalogram (EEG) tests conducted in 2002 and 2006 showed normal findings as well (Fig. 2).

#### Physical examinations and neuropsychological tests

His vital signs were stable, and he had clear lung sounds. There were no notable findings in neurologic examinations (sensation, muscle strength and cerebellar examinations). Neuropsychological tests such as Korean-Wechsler Adult Intelligence Scale (K-WAIS-IV), Rey-Kim memory test, Korean-Boston Naming Test (K-BNT) and Kims Frontal-executive Neuropsychological Test (K-FENT) were undertaken in January 2017. The results of the K-WAIS-IV test showed that his Full-scale Intelligence Quotient (FSIQ) was 68, which was in the extremely low level (the lowest 2.5%). In terms of sub-areas, he received a Verbal Comprehension Index (VCI) of 102, which corresponds to the average level (the lowest 23.6–73.8%), whereas a Perceptual Reasoning Index (PRI) of 68, a Working Memory Index (WMI) of 66 and a Processing Speed Index (PSI) of 55 were found to be in the extremely low level. The results of the Korean version Boston Naming Test (K-BNT) showed a normal level with 55 questions answered out of 60. As for concentration, the processing speed was found to be in the extremely low level, while the auditory, arithmetic and visual concentrations were found to be in the borderline level (the lowest 2.5–9.6%). In Rey-Kim memory test, he received a memory quotient (MQ) of 49, which was in the extremely low level, and he was found to have the extremely low level of immediate recall, delayed recall and recognition as well. His Executive Function Quotient (EFQ) of 56 was also in the extremely low level. In terms of the perceptual motor ability, his visual reasoning and organizing abilities had been lowered along with the decreased ability to copy figures.

The tests such as K-WAIS-IV test and Rey-Kim memory test were performed at Inje University Hospital in May 2014. In the K-WAIS-IV test, he had an FSIQ of 74, which is in the borderline level. In terms of sub-areas, he had a VCI of 102, a PRI of 78, a WMI of 75 and a PSI 55. It is found that his abilities of time orientation, sustained attention, verbal memory (long term), calculation, motor inhibition, motor set shifting and interference control had been identified as the extremely low level. The oldest data showing the patients’ cognitive function status was the 2002 epidemiological investigation report. The report did not include exact test methods and figures, and only an approximate level of cognitive function could be confirmed from it. According to the 2002 report, in terms of cognitive function, his FSIQ, visual sensitivity and attention fell in the low average level; his memory was in the borderline level; and his psychomotor speed fell in the extremely low level.

Although the difference in institutions and test methods interrupts an exact comparison, a rough comparison can be made with the tests performed in different years. His FSIQ was in the low average level in 2002, and it decreased to the borderline level in 2014 after four years of working as a shipyard painter and further to the extremely low level in 2017. His memory was in the borderline level in 2002, and it decreased to the extremely low level in 2017. His attention was in the low average level in 2002 and it decreased to the extremely low level in 2017. From these, it can be found that his cognitive function had gradually worsened.

## Discussion and conclusion

It is well known that neuropsychological diseases can occur among workers exposed to organic solvents. Organic solvents are contained in paint, thinner, hardening agent, polish, adhesive and degreasing agent, and the exposure to organic solvents can occur while painting, printing, polishing and coating works are performed [4, 8]. Work-related exposure to organic solvents generally occur through inhalation of vapor or dermal contact. And the organic solvents absorbed in the human body can affect tissues with a lot of fat, such as brain and nerve tissues, because they have lipophilic character. A long-term exposure to low-dose organic solvents can cause decreased cognitive function such as concentration disorder and memory disorder; affective disorders including anxiety, fatigue and depression; and movement disorders. These are referred to as symptoms of chronic toxic encephalopathy [9, 10].

World Health Organization (WHO) and ISW presented classifications for CTE, and the classifications can be referenced for diagnosis and determination of progression [6, 11]. According to the ISW classification, Type 1 has only nonspecific symptoms such as fatiguability, memory impairment, difficulty in concentration, and loss of initiative. These symptoms are reversible if exposure is discontinued, and there is no objective evidence of neuropsychiatric dysfunction. Type 2A is associated with a sustained personally or mood change, there is a marked and sustained change in personality involving fatigue, emotional lability, impulse control, and general mood and motivation. Type 2B is that symptoms continues to impairment in intellectual function, there is difficulty in concentration, impairment of memory and a decrease in learning capacity. Finally, Type 3 is dementia stage, in this condition, marked global deterioration in intellect and memory is often accompanied by neurological signs and neuroradiological findings. The diagnosis of CTE can be made by (1) the identification of a chronic exposure to a considerable amount of neurotoxic organic agents; (2) the confirmation of the clinical image of the damaged central nervous system through subjective symptoms and objective tests; and (3) the exclusion of other diseases through differential diagnosis [6, 11–13].

### The extent of chronic exposure to neurotoxic organic solvents

A painter is an occupation with a lot of exposure to organic solvents, and also, an occupation with the highest incidence of CTE in Finland. In South Korea as well, it shows a high risk of CTE since five out of a total of 10 workers who have obtained industrial accident approval are painters [3, 4]. The patient of this case worked as a shipyard painter for a total of 20 years, 15 years before the industrial accident approval and five years after the industrial accident approval. The paint and thinner that shipyard painters use are frequently changed; therefore it is difficult to find out exactly about the toxic substances to which they are exposed. However, in South Korea, the most commonly used organic solvent in paint is xylene, which is contained in about 77% of paint products (21% xylene by volume on average). As for thinners, it is reported that xylene is contained in about 60% of thinner products and toluene in about 22% of thinner products [14]. Consequently, it is confirmed that a considerable amount of aromatic hydrocarbon with high neurotoxicity is contained in paint and thinner products [15].

In this case, the mixed solvent index was used for the exposure evaluation, but it was impossible to make an objective judgment of the exposure during the period from when he started painting work at the shipyard to the first half of 1997, since the data from the period were not available. The measurements during the period from the second half of 1997 to the first industrial accident approval were found to be within the standard value. However, it can be assumed that the patient was exposed to a large amount of organic solvents during the period according to his statement that he did not wear appropriate protection gear from time to time and that he often worked more than ten hours a day. When he returned to work as a shipyard painter after the industrial accident approval, the exposure to mixed organic solvents was mostly found to be over the standard value. Therefore, it can be presumed that he was exposed to a considerable amount of organic solvents even after his return to work. In addition, it is known that even the exposure within the standard value can cause neuropsychological damage [16, 17]. In short, the patient of this case is found to have a history of exposure to organic solvents for over 20 years and a frequent exposure to the organic solvents above the standard value can be assumed especially after he returned to work following the diagnosis of CTE.

### Identification of neuropsychological damage

With regard to CTE, the mechanisms of neurotoxicity have not been clearly determined and there are many non-specific cases in terms of diagnostic radiology; therefore, neuropsychological tests play a key role in identifying neuropsychological damage [13]. The results of the neuropsychological tests performed in 2017 show that his FSIQ were in the extremely low level and severe neuropsychological damage was thus identified. And a large decrease in cognitive function was found compared to 2002. In terms of sub-areas, his ability in the linguistic area was found to correspond to the normal level, whereas his memory, concentration and executive function are found to be in the disorder level. Such an aspect of decreased cognitive function is consistent with the systemic review that the decreased cognitive function of CTE is most severe in the memory and attention areas and is relatively spared in the linguistic area.

The brain MRI performed in 2002, 2006 and 2017 showed normal findings, and this was consistent with the results of the existing research in which the brain MRI could showed normal findings regarding CTE [18–20]. Also, the EEG tests performed in 2002 and 2006 showed normal findings; this was consistent with the results of the existing research in which EEG tests could showed non-specific findings regarding CTE [21].

### Differential diagnosis

Diseases that need differential diagnosis for exclusion include non-occupational neuropsychological diseases (such as Alzheimer’s diseases and Parkinson’s diseases), infection diseases, inflammation diseases, neoplasm, paraneoplastic syndromes, traumatic brain injury, Metabolic disorders that can affect cognitive function, psychological cause, sleep disorders, and alcohol or substance abuse [13]. It is known that the depression of the patient can cause decreased cognitive function. And the general tendency of cognitive impairment by depression is mainly in the recall tasks because they require a lot of efforts, whereas the decrease in recognition tasks is minor [22]. However, in this case, the results of the memory test show the patient’s recall and recognition were both worsened to the extremely low level; therefore, the decrease in his cognitive function is considered different from that of depressions. In addition, he had a history of being diagnosed with organic solvent-induced depression. Consequently, depression is not an appropriate disease for differential diagnosis. With regard to Alzheimer’s disease, another disease subject to differential diagnosis, it can generally cause a disorder in naming ability [23]. The results of the K-WAIS-IV test performed in 2017 show that the linguistic ability of this patient was relatively spared, and he was also found to have an excellent level of naming ability. Consequently, there is a low possibility of Alzheimer’s disease. Alcohol-induced disorders in cognitive function are often accompanied by peripheral neuropathy or cerebellar ataxia, which are not identified in this case. And alcohol-induced disorders in cognitive function are known to result from an intake of over 60 g of alcohol per day. The patient in this case had an intake of less than 10 g of alcohol per day, a negligible amount, and he had not drunk alcohol since 2001. Consequently, there is a low possibility of an alcohol-induced disorder in cognitive function. Underlying brain diseases such as neoplasm and traumatic injury were excluded through MRI tests and EEG tests, and systemic diseases such as metabolic disease, liver disease and inflammatory disease were excluded through blood tests. Consequently, the possibility of diseases other than CTE was excluded when it comes to diseases that can cause decreased cognitive function, and the decrease in the patient’s cognitive function is considered to result from organic solvents.

This case is showing that CTE can go on without appropriate measures. At the time of industrial accident approval in 2001, the patient in this case was categorized as CTE ISW type 2A, whose main symptom is depression. Currently, his CTE has progressed into ISW Type 3, which is accompanied by a severe impairment in cognitive function. “Fitness to work” is an assessment of whether specific tasks are appropriate for an employee to perform such tasks without the employee or his colleagues’ health being affected by such tasks. It is well known that CTE is a non-progressive disorder [7, 24], and the progression of his CTE could have been prevented if the patient had not returned to work as a painter.
